# The potential key genes and pathways associated with Wilms tumor in quest of proper candidates for diagnostic and therapeutic purposes

**DOI:** 10.1038/s41598-022-22925-3

**Published:** 2022-10-25

**Authors:** Masoud Bitaraf, Mohammadamin Mahmanzar, Narges Zafari, Hadiseh Mohammadpour, Mohammad Vasei, Leyla Moradi Matin, Abdol-Mohammad Kajbafzadeh, Masoumeh Majidi Zolbin

**Affiliations:** 1 Pediatric Urology and Regenerative Medicine Research Center, Gene, Cell and Tissue Research Institute, Children’s Medical Center, Tehran University of Medical Sciences, No. 62, Dr. Qarib’s St, Keshavarz Blvd, Tehran, 14194 33151 Iran; 2 Department of Bioinformatics, Kish International Campus, University of Tehran, Kish, Iran; 3 Department of Medical Genetics, School of Medicine, Tehran University of Medical Sciences, Tehran, Iran; 4 Dental Research Center, Dentistry Research Institute, Tehran University of Medical Sciences, Tehran, Iran; 5 Cell Therapy Based Research Center, Digestive Disease Research Institute, Shariati Hospital, Tehran University of Medical Sciences, Tehran, Iran

**Keywords:** Cancer, Genetics, Systems biology, Biomarkers, Oncology, Urology

## Abstract

To designate the probable most important differentially expressed genes and genetic pathways in Wilms tumor and assess their expression and diagnostic potential by RT-PCR and statistical analysis. Systematic review of the literature and various bioinformatics analysis was carried out to gather and narrow down data. The expression of end-resulting genes was compared in Wilms tumor and normal tissue samples using RT-PCR. Statistical tests reported the diagnostic accuracy of genes and their correlation with clinicopathological features. Four genes including CDH1, NCAM1, EGF, and IGF2 were designated. The panel combining them has 100% sensitivity and specificity in differentiating tumors from normal tissue. Eight pathways, most involved in cell–cell and cell-basal matrix junction interactions, were found to be associated with disease pathogenesis. The suggested genes should undergo further evaluation to be validated as diagnostic biomarkers. Further research on the eight proposed pathways is recommended.

## Introduction

The most prevalent pediatric renal malignancy, Wilms tumor (WT), occurs with an incidence of 3–10 cases per million, varying between different ethnicities. It forms due to aberrant nephrogenesis secondary to sporadic or hereditary mutations in genes involved in the process^1^. Despite a long-term survival rate of more than 85%, three subgroups of patients, including those with bilateral, relapsed, or anaplastic tumors, are confronted by treatment challenges^2^. Moreover, although pathology remains the gold standard in diagnosing and identifying biological features of tumors; obtaining tissues requires invasive methods that are costly, painful, and difficult to perform in children. Furthermore, advanced imaging techniques, used to diagnose and monitor disease, expose the children to radiation and/or anesthetic drugs^3–5^. WT is associated with various genetic changes identification of which yields to diagnostic, prognostic, and therapeutic advances. Studying the genetic basis and differentially expressed genes (DEGs) are of great value in identifying disease biomarkers and pathogenesis, which results in the development of liquid biopsy assays to diagnose and monitor response to treatment. A better understanding of pathogenesis could also lead to identifying novel therapeutic targets and advances toward personalized medicine^4–7^. Due to progressions of high-throughput techniques, big data is available on genomics, proteomics, and metabolomics regarding different diseases. We investigated through extensive data available in the literature and Gene Expression Omnibus (GEO) database, in a struggle to propose potential candidates as markers of early WT detection, possible targets for novel treatments, and/or indicators of response to treatment. We designated 4 genes in this regard and further analyzed the diagnostic potential of these genes as biomarkers for disease detection. We also aimed to introduce a limited number of pathways, worth studying according to our valid findings, to other researchers who wish to work on WT. This paper aims to describe how a long list of genes associated with WT are narrowed down to main candidates for diagnostic purposes by applying bioinformatics-based analysis and systematic review of the literature. They were then studied in the laboratory using relative comparative RT-PCR. Statistical analysis revealed their capacity to differentiate between WT patients and healthy people. Another objective pursued by this paper is to provide the legitimate reader with leads and questions to follow and answer to unravel the unknowns of WT.

## Materials and methods

### In silico and literature studies (Fig. 1)

**Figure 1:**
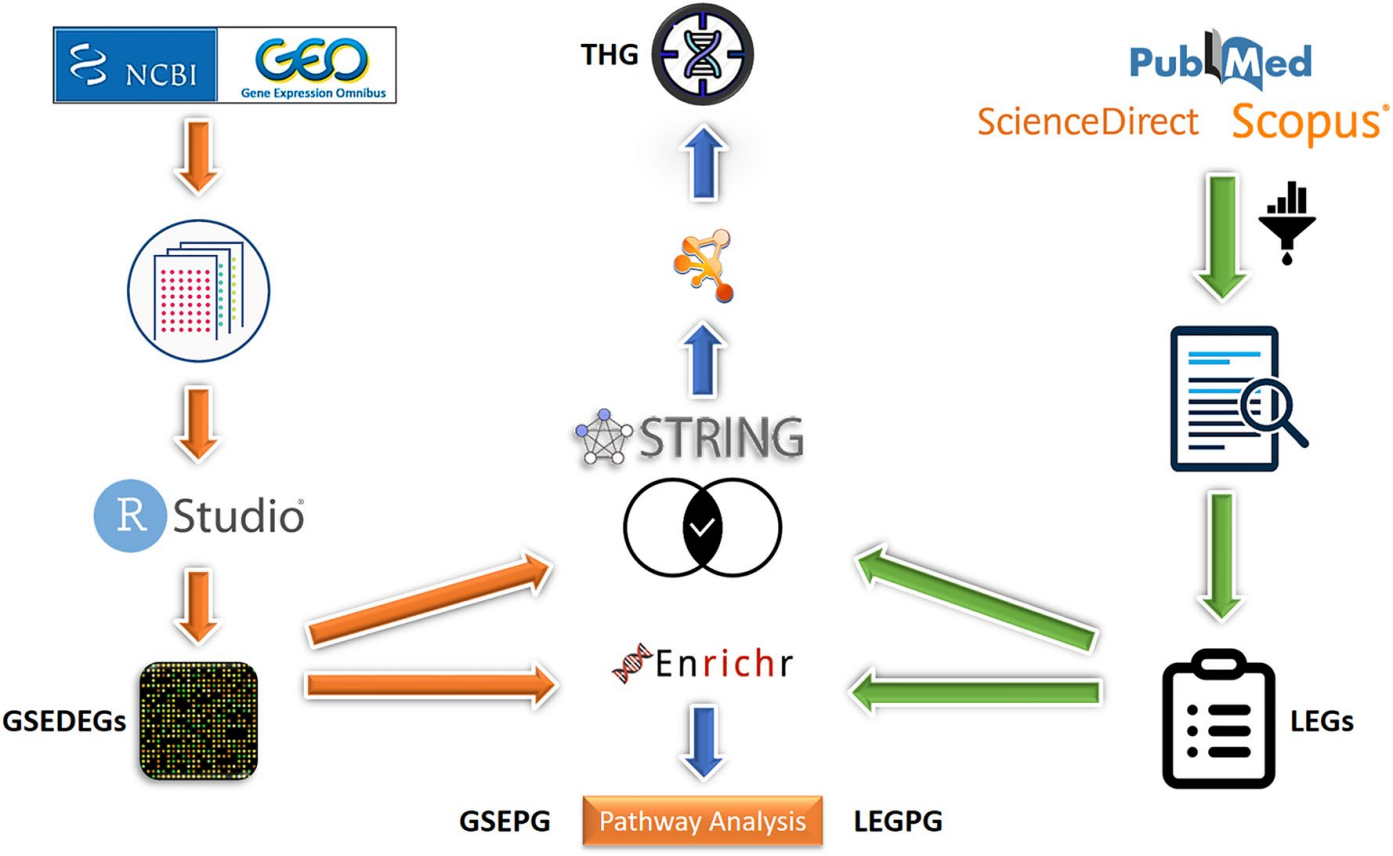
Graphical abstract of ‘in silico and literature studies’ section of methods and material. LEGs, literature extracted genes; GSEDEGs, GSE differentially expressed genes; THG, top 100 hub genes; LEGPG, LEGs pathways genes; GSEPG,GSE pathways genes.

#### Data collection

The expression of genes involved in Wilms tumor (WT) was inquired of two separate sources in April 2020.

"Wilms tumor" and "gene expression" keywords were used to search NCBI PubMed, Elsevier, Embase, and Scopus databases. With no filter applied, all articles were imported (our search strategy in Pubmed is provided in Table 1 as an example). After removing duplications, 2199 articles were included for the primary survey using title and abstract to extract articles comparing tissue samples of healthy humans to patients with WT and remove remainders. Subsequently, the remaining articles were reviewed, and various genes with different expressions in WT and p-value < 0.05 were extracted to form a list called "Literature Extracted Genes" (LEGs).

**Table 1:** Search strategy details.

(("wilms tumour"[All Fields] OR "wilms tumor"[MeSH Terms] OR ("wilms"[All Fields] AND "tumor"[All Fields]) OR "wilms tumor"[All Fields]) AND ("gene expression"[MeSH Terms] OR ("gene"[All Fields] AND "expression"[All Fields]) OR "gene expression"[All Fields]))	

Using the same keywords, National Center for Biotechnology Information (NCBI) GEO database was searched. Results were filtered by "Homo sapiens" as organism, "expression profiling by array" as study type, and "tissue" as the attribute name. The results were then examined to compare a healthy human with a patient with WT; hence selection of "GSE66405" dataset. This dataset comprises mRNA expression profiles of 32 tissue samples (28 WT and 4 normal) examined by the "GPL17077 Agilent-039494 SurePrint G3 Human GE v2 8×60K Microarray" platform. This dataset is called "GSE" throughout the manuscript.

#### Data analysis

**Figure 2:**
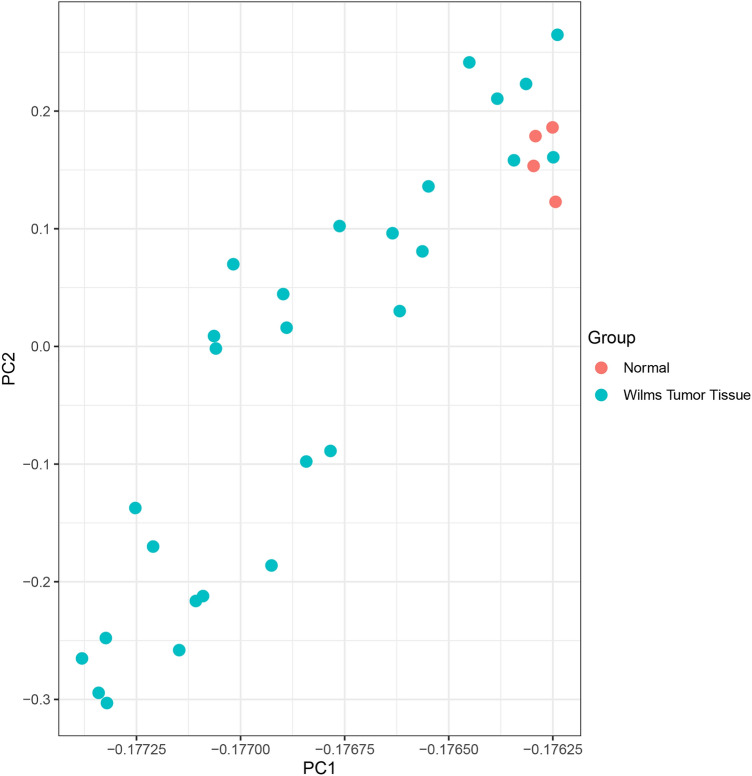
PCA analysis. Principal component analysis (PCA) of GSE data shows that all data were imported in 2 dimension and normal group represents a cluster with minimum laboratory error.

**Figure 3:**
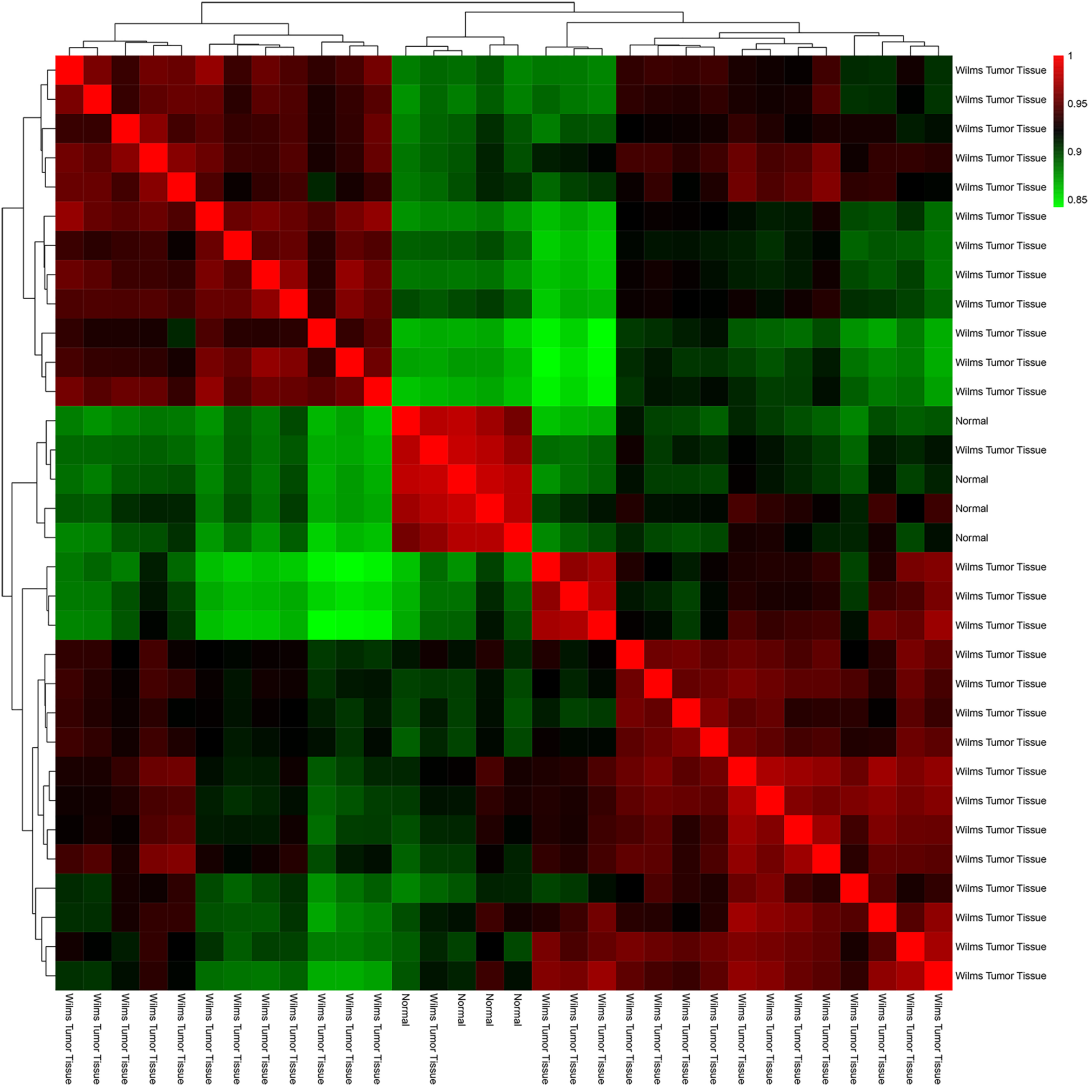
GSE DATA correlation heatmap shows correlation between tumor group versus normal group.

**Figure 4:**
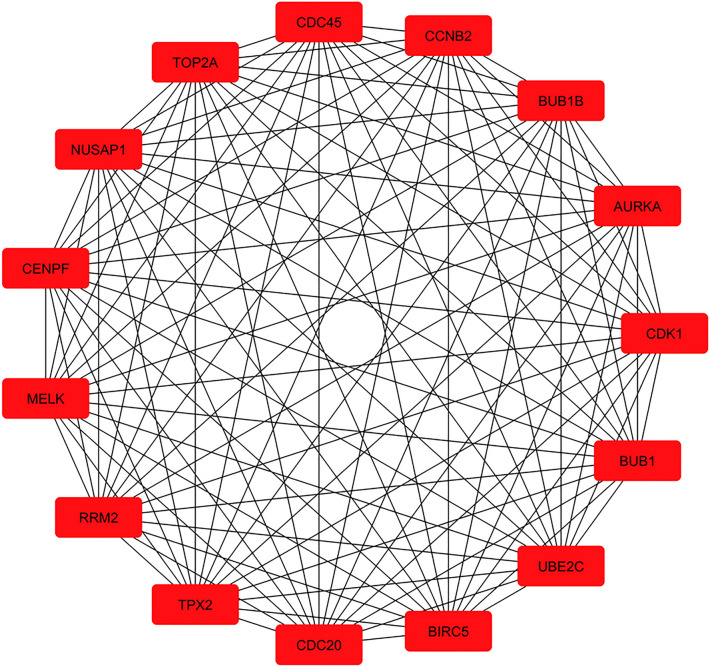
Top 15 genes from 100 mixed literature and GSE ranked genes in networks.

To characterize DEGs of the GSE dataset, it was imported into the R/Bioconductor platform (version Rx64 3.3) and normalized. The "ggplot" package was used to draw a graph that represents normalized data (Fig. 2). Moreover, a quality control check was performed on data using the "Pheatmap" package, demonstrating a correlation between samples (Fig. 3). Finally, multiple LIMMA (linear models for microarray data) package testing options were employed to identify DEGs within normalized values. For the sake of high statistical accuracy, gene expression fold change with a cut-off of 2 (LogFC > 2|logFC < − 2) and an adjusted p-value threshold of 0.05 was selected. It is essential to arrange genes in clusters and rank them to understand the correlation between genes and their importance in WT pathogenesis. To do so, a list of genes composed of GSE DEGs and LEGs was imported to the String database (ver:11.0, http://string.embl.de/), which plotted Protein–Protein Interaction (PPI) network. This information was then downloaded in TSV format and imported into Cytoscape 3.7.2 software to analyze the network and recognize genes of more probable importance. This software allows the calculation of various centrality parameters, including the degree and betweenness of each gene separately. CytoHubba plugin^8^ was then utilized to rank genes based on degree and betweenness. This study selected the first 100 genes as "Top 100 Hub Genes (THG)". PPI network of the top 15 among these 100 is illustrated in Fig. 4 as an example.

#### Genes' pathways

Genes can act separately and/or in companion with other genes as part of a pathway. LEGs and GSE DEGs were individually imported into the Enrichr database (https://amp.pharm.mssm.edu/Enrichr/). This database provides a wide variety of information on genes, including pathways associated with the input genes. Pathways with an adjusted p-value of less than 0.05 of each separate list were selected for further analysis. Genes involved in pathways associated with each list were then extracted to form another two lists named "LEGs Pathways Genes (LEGPG) and "GSE Pathways Genes (GSEPG)," respectively.

#### Data validation

Since microarray data were used in our analysis, TNMplot (https://tnmplot.com/) was used to validate our final candidate genes with RNA-seq data^9^.

### Histopathology and real-time PCR

#### Samples

Paraffin blocks of 12 WT patients stored from April 2019 to March 2021 in the children's medical center (CMC), Tehran, Iran, were collected. Two samples were acquired from each slide; tumor tissue and adjacent normal tissue. In accordance with the declaration of Helsinki based on relevant guidelines of Tehran University of Medical Sciences (TUMS), before all surgeries, informed consent prepared which was asking permission to use samples for research purposes as well as data publication and obtained from the patient's parents or guardians. This study is approved by the ethics committee of the CMC, TUMS with the following ethic code IR.TUMS.MEDICINE.REC.1400.523.

#### RNA extraction and cDNA synthesis

According to the manufacturer's instructions, samples were deparaffinized using xylene and were subjected to RNA extraction by RiboExTM (Geneall Biotech, Korea). RNA quantification was carried out via concentration and absorbance evaluation by NanoDrop™ 2000/2000c Spectrophotometers (Thermo Fisher Scientific, USA). The absorption ratio in 260/230 nm and 260/280 nm were assessed. The ratio between 1.8–2.2 and 1.7–1.9 were considered proper values, respectively. Synthesis of cDNA was executed using Reverse Transcription Reagents (BioFact™ OneStep RT-PCR). The products were used directly in qPCR or stored at − 20 °C. In cases of longer storage, cDNAs were stored at − 70 °C.

#### Relative comparative real-time PCR

Initially, Primers were synthesized for four candidate genes; NCAM, IGF2, EGF, and CDH1, according to the sequences collected from OriGene (https://www.origene.com). Relative comparative RT-PCR was carried out using LightCycler® 96 Instrument (Roche Life Science) following manufacturer's instructions (10 µL SYBR Green Master Mix, 10 pmol/µL forward and reverse primers each, 200 ng/µL template cDNA and distilled water to a total volume of 20 µL were entered each reaction). Expression values of mRNAs were obtained and compared with the control group.

#### Statistical analysis

Where appropriate, data were reported as mean, standard deviation (SD), or proportions. The student's t-test was used to compare the clinical characteristics of Wilms tumor patients to those of the control group. The Kolmogorov–Smirnov test was performed to determine the normal distribution of tumor-related variables. The expression levels of genes were compared between groups using the non-parametric Mann–Whitney U test for non-normally distributed data (P-value < 0.05) and the parametric t-test for normally distributed data (P-value > 0.05). To conduct clinicopathological correlation analysis, we used the median expression of genes as a cut-off value for categorizing 12 patients into two groups: those with relatively high expression and those with relatively low expression. The chi-square and Fisher exact tests compared clinicopathological features between genes with low and high expression levels. IBM SPSS Statistics 22 (SPSS Inc., Chicago, IL, USA) was used for statistical analysis. A P-value of 0.05 indicates that differences between groups are statistically significant. The receiver operating characteristic curve (ROC) analysis was used to estimate the diagnostic accuracy of gene expression for each gene alone and in two, three, and four combinations. The optimal statistical cut-off values for gene expression levels were determined, followed by the ROC curve's sensitivity and specificity of selected cut-off points. Bar charts were created in this work using GraphPad Prism 8 (GraphPad Software Inc., San Diego, CA, USA).

### Ethics approval and consent to participate

IR.TUMS.MEDICINE.REC.1400.523.

## Results

### In silico and literature

#### Genes and pathways

**Figure 5:**
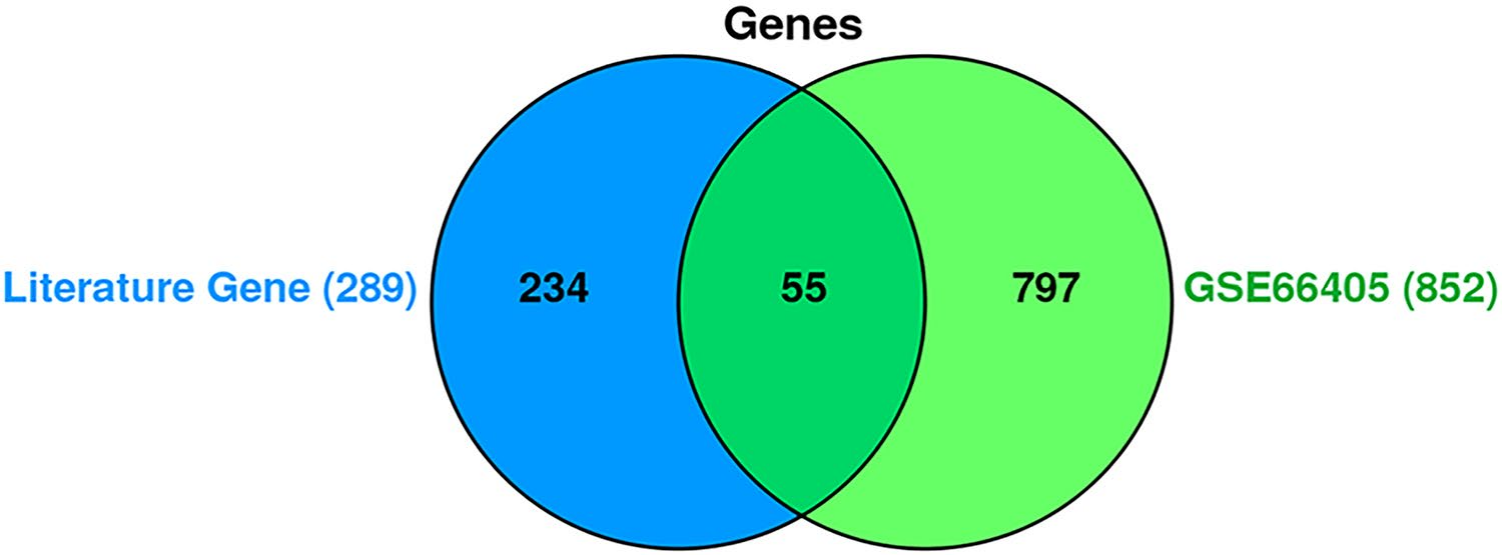
Common genes between literature genes and GSE analysis gene, showing 55 common genes.

**Figure 6:**
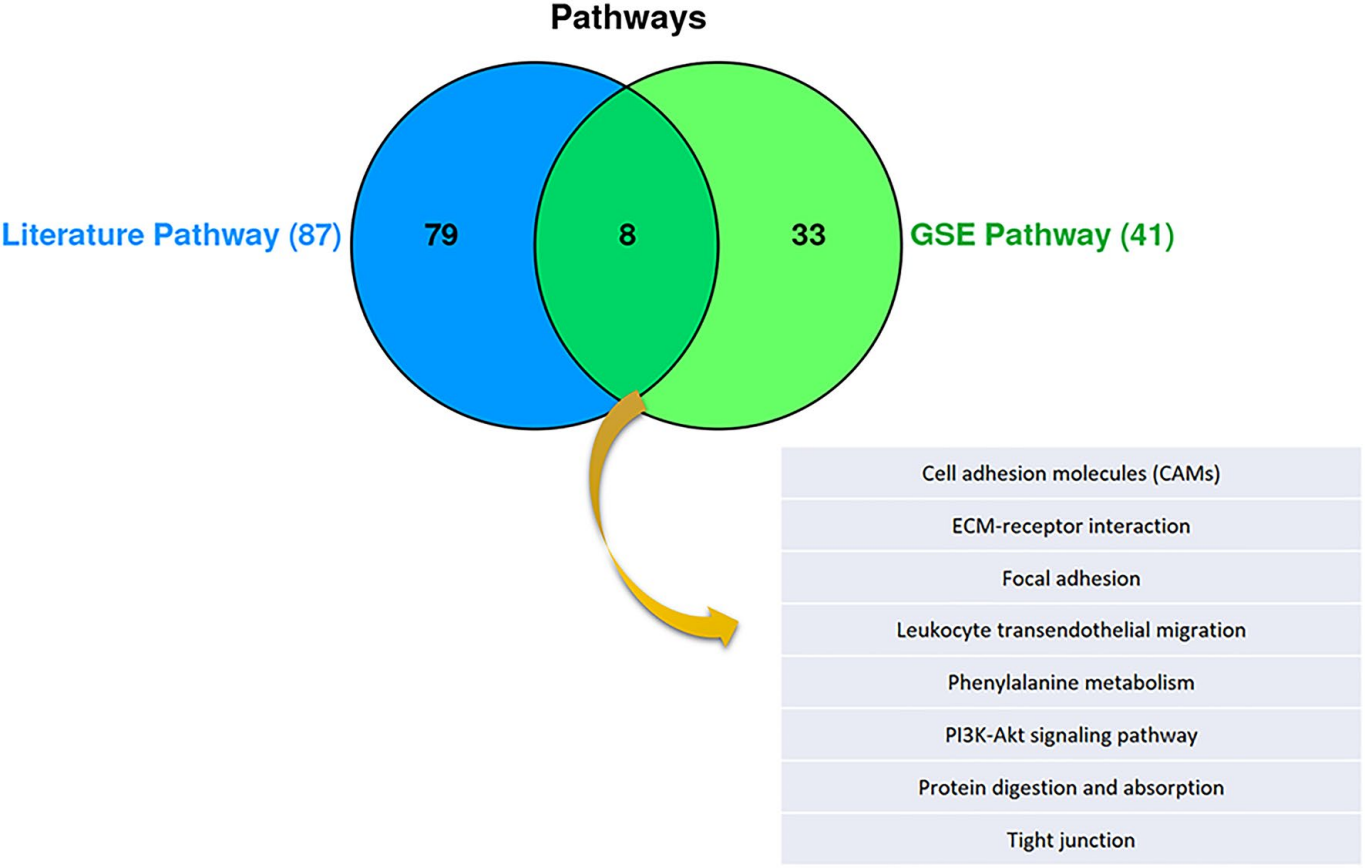
Pathway analysis between literature genes pathway and GSE genes pathways, show 8 common pathways between these.

**Figure 7:**
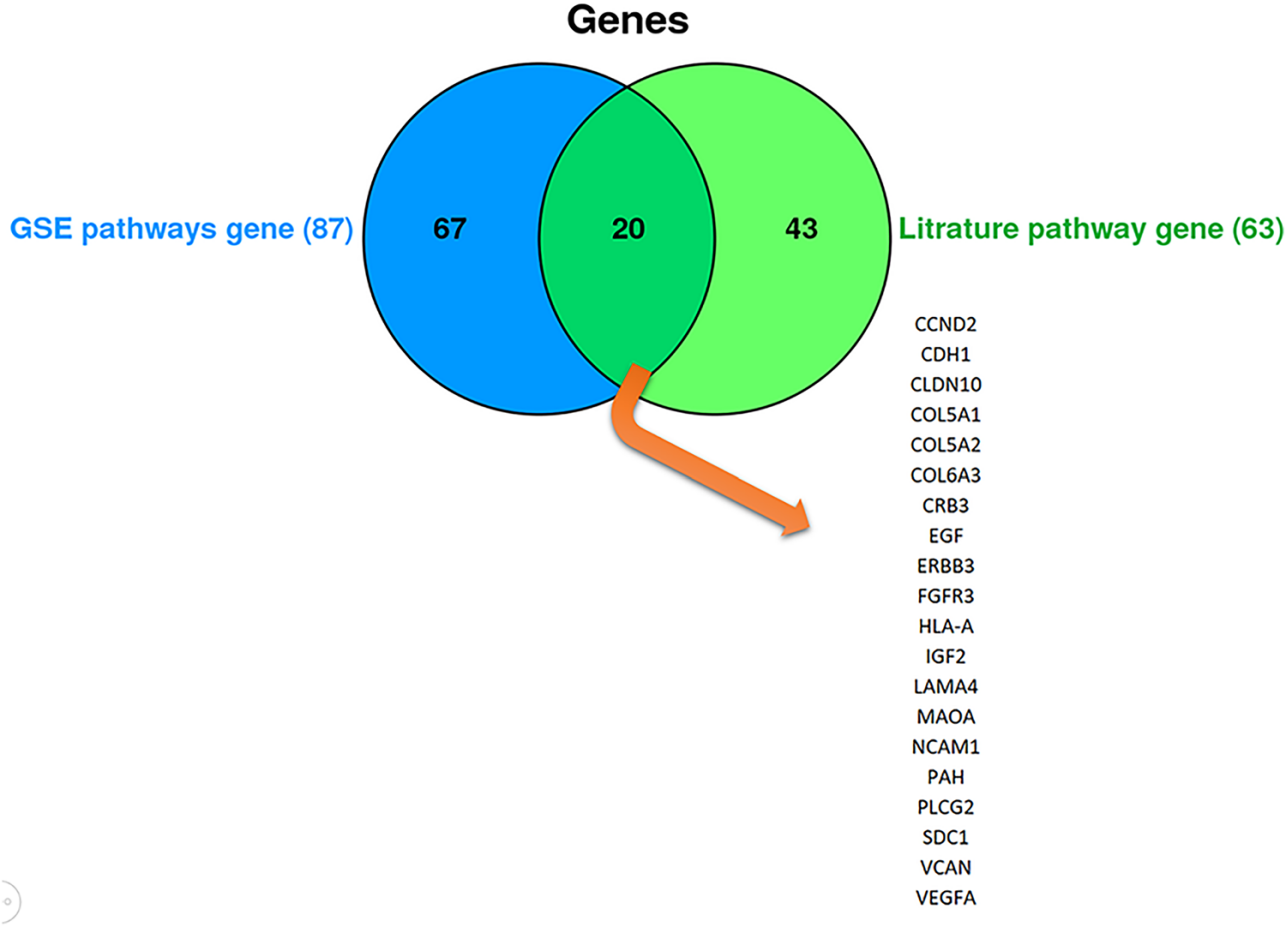
Common genes between GSE pathway and literature pathway.

**Figure 8:**
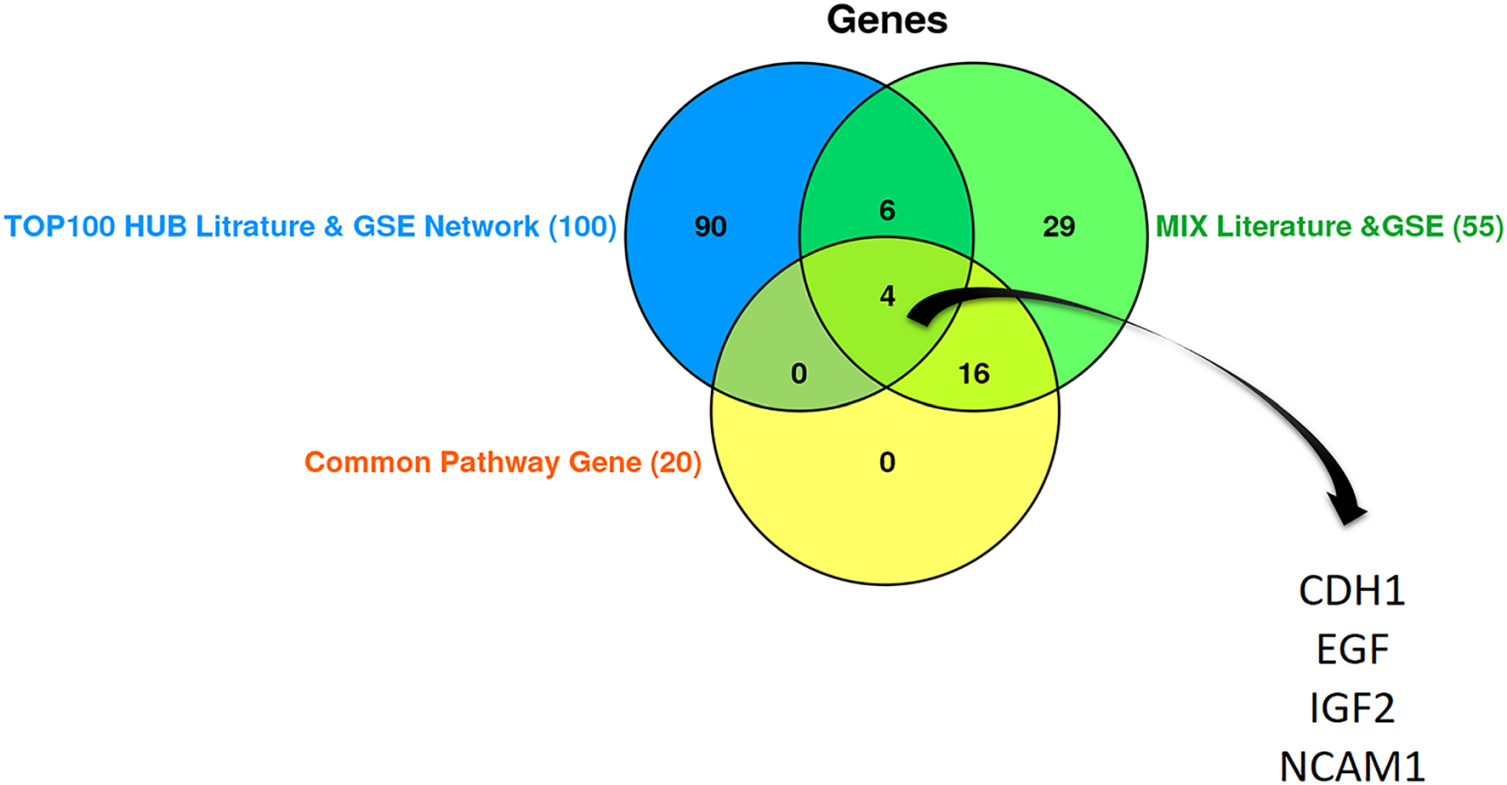
Diagram depicting 4 common genes among 3 lists.

The initial literature searches and duplication removal resulted in 2199 articles. From those, 59 articles (supplementary table 1) were included into the study after the primary survey by title and abstract. A total of 289 genes were extracted from these 59 articles as LEGs. Additionally, analysis on the GSE dataset yielded 852 genes with differential expression according to criteria mentioned earlier (GSE DEGs). These to lists shared 55 common genes that were labeled as "Common Genes Group A (CGA)" (Fig. 5). Enrichr database provided 87 pathways from LEGs and 41 from GSE DEGs, with a P-value < 0.05. Eight pathways were common among both (Fig. 6) consisting of: Cell adhesion molecules (CAMs), ECM-receptor interaction, focal adhesion, leukocyte transendothelial migration, Phenylalanine metabolism, PI3K-Akt signaling pathway, protein digestion and absorption, and tight junction. We compared LEGPG and GSEPG and found 20 common genes, and labeled them as "Common Genes Group B (CGB)" (Fig. 7). Finally, we put THG, CGA, and CGB together and found common genes among them (Fig. 8). As illustrated in Fig. 8, the results were narrowed down to four genes; CDH1, EGF, IGF2, and NCAM1. NCAM1 and IGF2 expression levels were upregulated with a Log FC = 4.339 and Log FC = 3.619, respectively. CDH1 and EGF showed downregulated expression with Log FC = − 3.850 and Log FC = − 6.476, respectively.

#### Data validation

**Figure 9:**
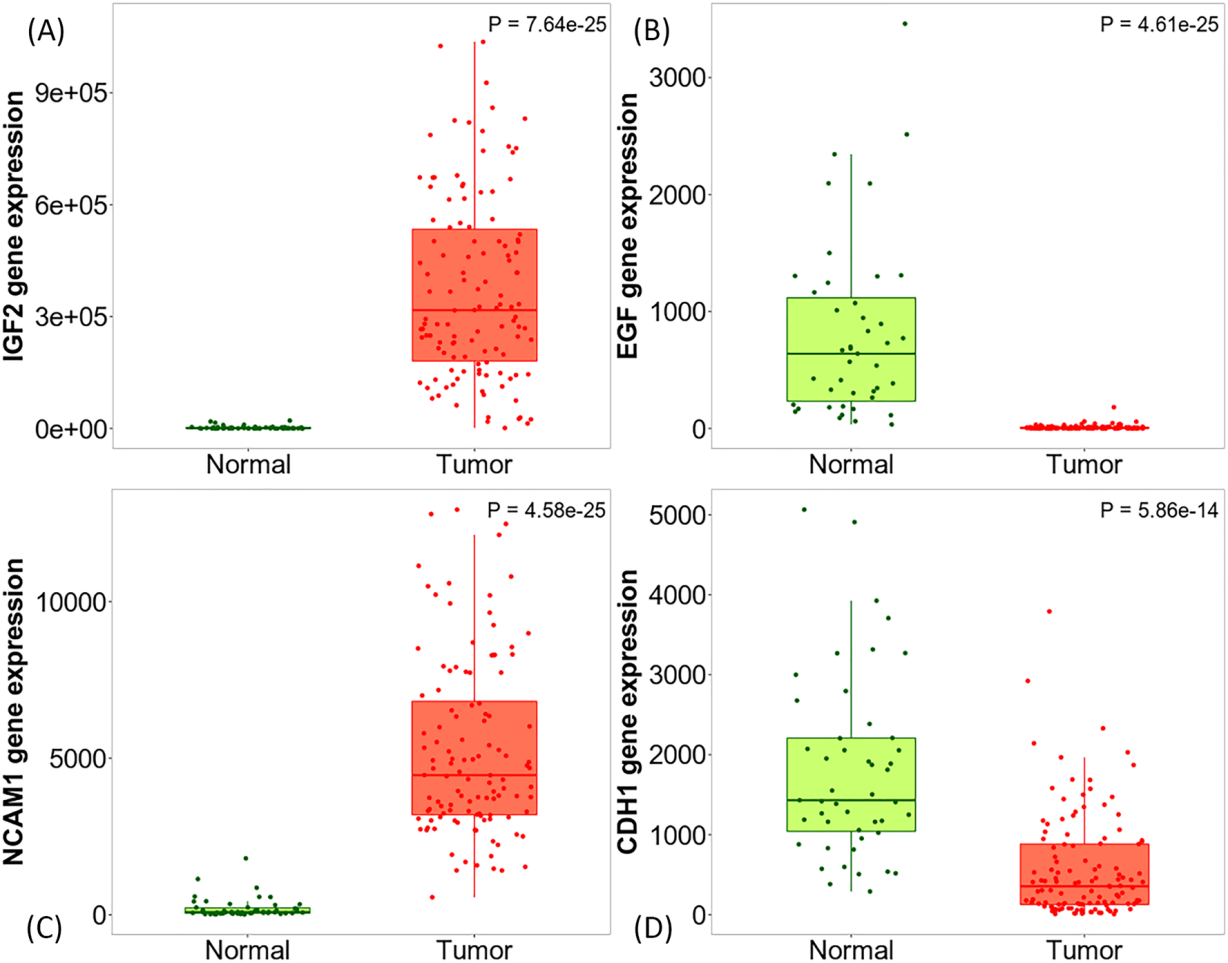
TNM plot of four candidate genes which are evaluated in RNA-seq technique. (**A**): IGF2, (**B**): EGF, (**C**): NCAM1, (**D**): CDH1.

TNMplot depicted the same differential expression pattern of four candidate genes in the RNA-seq technique (Fig. 9).

### Histopathology and real-time PCR

In this study we used paraffin blocks of 12 WT patients consisting of six girls and six boys with the mean age of 3.98 and 3.83, respectively. Samples from these blocks were obtained providing 12 WT tissue- and 12 adjacent normal tissue-samples. Table 2 summarizes the demographic data of patients and the pathologic features of their tumors.

**Table 2:** Demographic and pathologic data of cases. Null: not identified; Age is reported in the form of years + months.

Case number	Pathologic morphology	Histology	Lymph node involvement	Capsule involvement	Gerota's Fascia involvement	Perinephric fat involvement	Stage	Age	Sex
3819	Biphasic	Favorable	−	+	+	+	2.00	9 + 0	Male
1602	Triphasic	Favorable	−	+	+	+	2.00	4 + 8	Female
3560	Null	Favorable	+	Null	−	−	3.00	5 + 2	Male
2043	Monophasic	Favorable	+	+	+	+	3.00	3 + 6	Female
3197	Triphasic	Favorable	−	+	−	−	2.00	3 + 0	Male
4453	Triphasic	Favorable	Null	+	Null	Null	Null	6 + 4	Female
508	Triphasic	Favorable	−	+	+	+	2.00	4 + 2	Male
891	Triphasic	Favorable	−	+	+	+	4.00	5 + 11	Female
2628	Biphasic	Favorable	−	−	−	−	2.00	0 + 10	Female
232	Triphasic	Favorable	−	−	−	−	2.00	1 + 7	Male
1921	Biphasic	Favorable	+	+	+	+	3.00	1 + 0	Male
1173	Triphasic	Favorable	−	+	+	+	2.00	1 + 9	Female

#### Differential expression of NCAM1, IGF2, CDH1 and EGF in the tissue samples of Wilms Tumor and adjacent normal tissues

**Figure 10:**
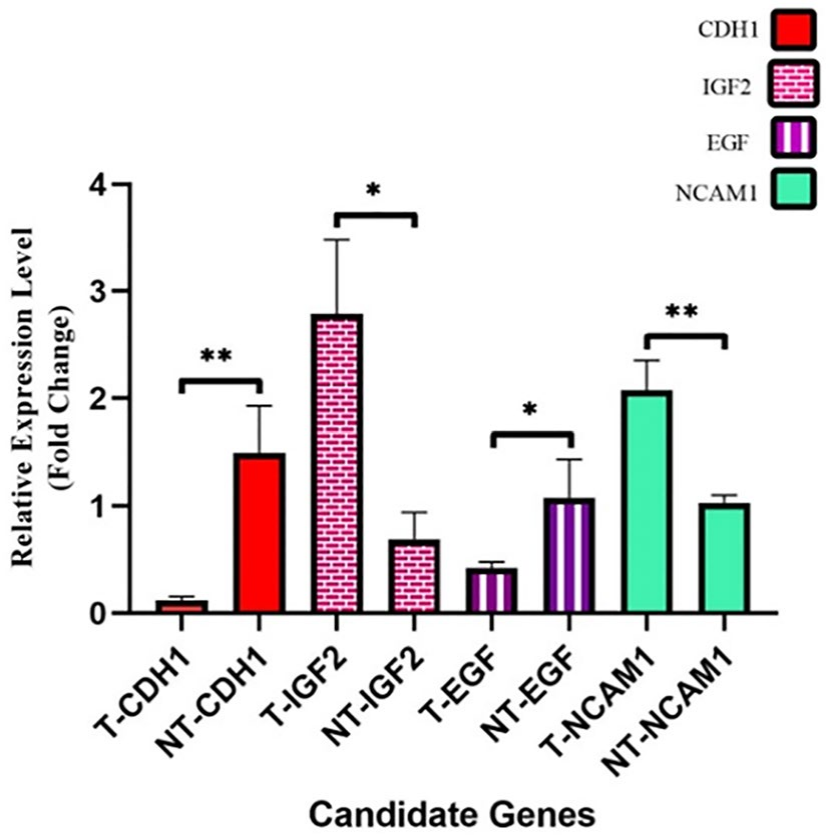
Relative expression of four candidate genes comparing tumor tissue with normal adjacent tissue.

RT-qPCR was performed to assess the relative level of NCAM1, IGF2, CDH1 and EGF in the tissue samples. In detail, the expression levels of NCAM1 and IGF2 were significantly higher in tumor tissue compared to adjacent normal tissue from the same patient (P-value of < 0.01 and < 0.05, respectively). In contrast, the expression level of CDH1 and EGF (P-value of < 0.01 and < 0.05, respectively) were significantly lower in the tumor tissues group rather than those found in adjacent normal tissues (Fig. 10).

#### Differential expression of NCAM1, IGF2, CDH1 and EGF based on the disease stages and patients’ gender

**Figure 11:**
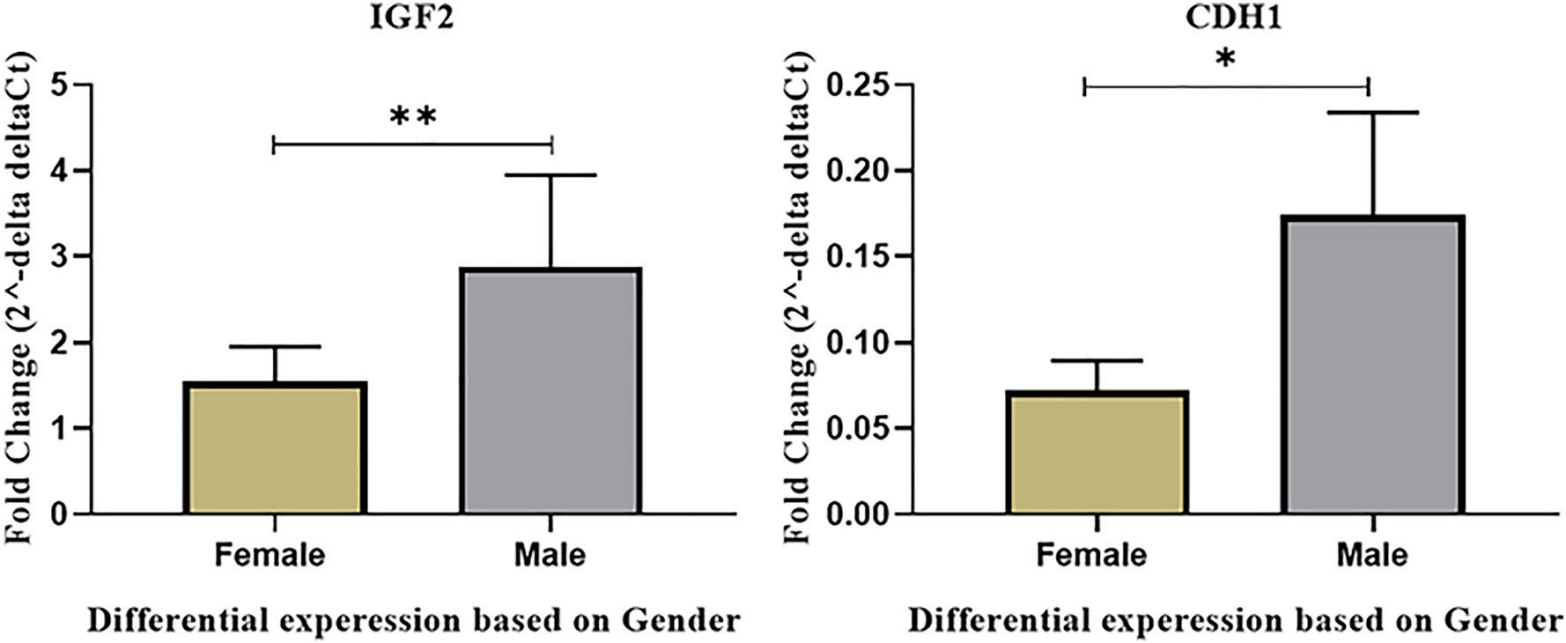
Differential expression of (**A**) IGF2 and (**B**) CDH1 based on gender (P value*: P value < 0.05, **: P value < 0.01, ***: P value < 0.0001).

We tested the association between NCAM1, IGF2, CDH1 and EGF expression levels and the specific stage of disease of the study participants. There was no significant association between NCAM1, IGF2, CDH1 and EGF expression levels with different stages of WT. We also analyzed the association between the expression levels of these genes and the gender of included patients. There was no significant association between the expression levels of NCAM1 and EGF and the gender of patients. However, the expression level of IGF2 and CDH1 was significantly associated with the patient’s sexuality as the expression of both genes was higher in men (P-value < 0.05) (Fig. 11).

#### Correlation between NCAM1, IGF2, CDH1 and EGF expression and different histopathological features of patients

We also assessed the correlation between each gene expression with different demographic and histopathological features of patients such as age; pathologic morphology (Monophasic, Biphasic, and Triphasic); and the involvement of lymph nodes, renal capsule, Gerota Fascia, and peri-nephric fat. As shown in Table 3, there was no significant correlation between high- or low-expression of NCAM1, IGF2, CDH1, and EGF and with the above-mentioned features of patients (P-value > 0.05).

**Table 3:** Correlation of genes of interest’s expression with clinic-pathological features in Wilms tumor.

Characteristic	Case (n)	CDH1 expression		*P*-value	IGF2 expression		*P*-value	EGF expression		*P*-value	NCAM1 expression		*P*-value
		Low (n = 6)	High (n = 6)		Low (n = 6 )	High ( n = 6 )		Low (n = 6)	High (n = 6)		Low (n = 6)	High (n = 6)	
**Age at diagnose**				0.24			0.24			1.000			1.000
≤ 3	6	2 (33.3)	4 (66.6)		4 (66.6)	2 (33.3)		3 (50%)	3 (50%)		3 (50%)	3 (50%)	
≥ 4	6	4 (66.6)	2 (33.3)		2 (33.3)	4 (66.6)		3 (50%)	3 (50%)		3 (50%)	3 (50%)	
**Pathologic Morphology**				0.48			0.48			0.30			0.48
Null	1	1 (16.6%)				1 (16.6%)			1 (16.6%)			1 (16.6%)	
Monophasic	1	1 (16.6%)				1 (16.6%)			1 (16.6%)			1 (16.6%)	
Biphasic	3	1 (16.6%)	2 (33.3%)		2 (33.3)	1 (16.6%)		1 (16.6%)	2 (33.3)		2 (33.3)	1 (16.6%)	
Triphasic	7	3 (50%)	4 (66.6)		4 (66.6)	3 (50%)		5 (83.3)	2 (33.3)		4 (66.6)	3 (50%)	
Lymphnode In.*				0.40			0.40			0.10			0.51
Null	1	1 (16.6%)				1 (16.6%)		1 (16.6%)			1 (16.6%)		
Negative	8	3 (50%)	5 (83.3)		5 (83.3)	3 (50%)		5 (83.3)	3 (50%)		4 (66.6)	4 (66.6)	
Positive	3	2 (33.3)	1 (16.6%)		1 (16.6%)	2 (33.3)			3 (50%)		1 (16.6%)	2 (33.3)	
**Capsule**				0.21			0.21			0.21			0.21
Null	1	1 (16.6%)				1 (16.6%)			1 (16.6%)			1 (16.6%)	
Negative	2		2 (33.3)		2 (33.3)			2 (33.3)			2 (33.3)		
Positive	9	5 (83.3)	4 (66.6)		4 (66.6)	5 (83.3)		4 (66.6)	5 (83.3)		4 (66.6)	5 (83.3)	
**Gerota Facia In.**				0.34			0.34			0.56			0.56
Null	1	1 (16.6%)				1 (16.6%)		1 (16.6%)			1 (16.6%)		
Negative	4	1 (16.6%)	3 (50%)		3 (50%)	1 (16.6%)		2 (33.3)	2 (33.3)		2 (33.3)	2 (33.3)	
Positive	7	4 (66.6)	3 (50%)		3 (50%)	4 (66.6)		3 (50%)	4 (66.6)		3 (50%)	4 (66.6)	
**Perinepheric Fat In.**				0.34			0.34			0.56			0.56
Null	1	1 (16.6%)	3 (50%)		3 (50%)	1 (16.6%)		1 (16.6%)	2 (33.3)		1 (16.6%)	2 (33.3)	
Negative	4	1 (16.6%)	3 (50%)		3 (50%)	1 (16.6%)		2 (33.3)	4 (66.6)		2 (33.3)	4 (66.6)	
Positive	7	4 (66.6)				4 (66.6)		3 (50%)			3 (50%)		
**Stage**				0.30			0.48			0.09			0.48
Null	1	1 (16.6%)				1 (16.6%)							
II	7	2 (33.3)	5 (83.3)		4 (66.6)	3 (50%)		1 (16.6%)	2 (33.3)		1 (16.6%)	3 (50%)	
III	3	2 (33.3)	1 (16.6%)		1 (16.6%)	2 (33.3)		5 (83.3)	3 (50%)		4 (66.6)	2 (33.3)	
IV	1	1 (16.6%)			1 (16.6%)				1 (16.6%)		1 (16.6%)	1 (16.6%)	
**Sex**				0.24			0.24			0.24			1.000
Female	6	4 (66.6)	2 (33.3)		2 (33.3)	4 (66.6)		4 (66.6)	2 (33.3)		3 (50%)	3 (50%)	
Male	6	2 (33.3)	4 (66.6)		4 (66.6)	2 (33.3)		2 (33.3)	4 (66.6)		3 (50%)	3 (50%)	
**Age (mean)**													
4 year	12	–	–	0.28	–	–	0.27	–	–	0.28	–	–	0.28

*In.: Involvement.

#### Evaluation of the diagnostic performance of NCAM1, IGF2, CDH1 and EGF separately and in various combinations

**Figure 12:**
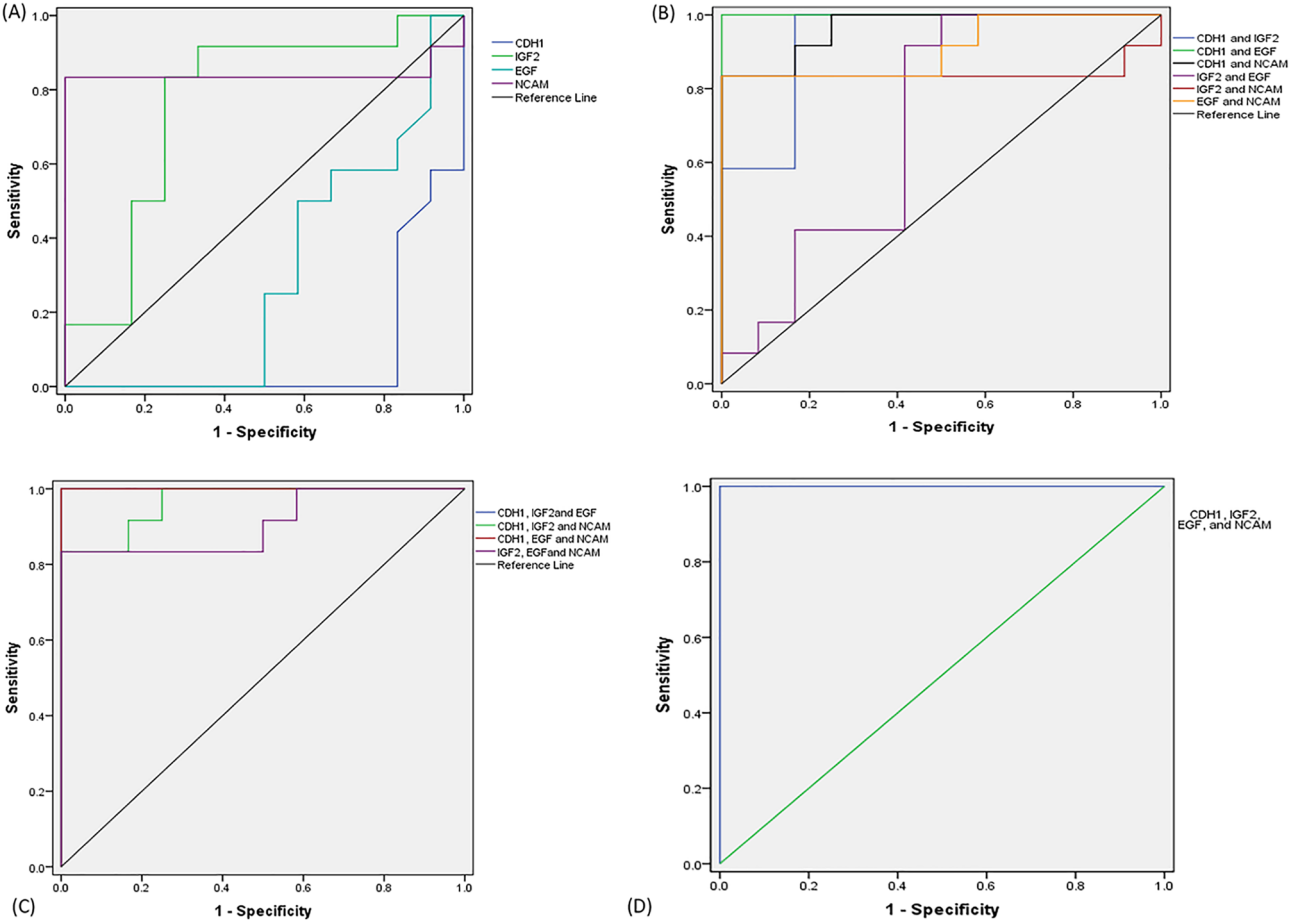
The results of ROC curve analysis for the diagnostic value of genes of interest (**A**) separately, (**B**) in binary combination, (**C**) ternary combination, and (**D**) quadric combination.

To estimate the performance of the identified genes for Wilms tumor diagnosis, we performed ROC curves analysis. The diagnostic accuracy of NCAM1, IGF2, CDH1 and EGF was measured by AUC which was 0.84 (sensitivity = 83% and specificity = 100%), 0.764 (sensitivity = 91% and specificity = 67%), 0.087 (sensitivity = 41% and specificity = 17%) and 0.302 (sensitivity = 58% and specificity = 34%), respectively. Based on the calculated AUC values, NCAM1 and IGF2 transcript levels had the fair performance in differentiation of patients from total controls (Table 4 and Fig. 12). Furthermore, the diagnostic accuracy of the different combinations of NCAM1, IGF2, CDH1 and EGF was reported and summarized in Table 4. The combinations of CDH1 and EGF, CDH1, IGF2 and EGF, CDH1, EGF and NCAM, and CDH1, IGF2, EGF and NCAM1 are able to discriminate patients from the control group with the AUC of 1.00 (sensitivity = 100% and specificity = 100%).

**Table 4:** Diagnostic accuracy of genes of interest separately and as a combined panel.

Gene of interest	AUC (area under curve)	Sensitivity (%)	Specificity (%)	Cut-off
CDH1	0.087	41	17	0.10
IGF2	0.764	91	67	0.75
EGF	0.302	58	34	0.35
NCAM1	0.84	83	100	1.43
**Specific combination**				
CDH1 and IGF2	0.93	100	84	0.37
CDH1 and EGF	1.00	100	100	0.50
CDH1 and NCAM1	0.96	91	84	0.43
IGF2 and EGF	0.70	91	59	0.49
IGF2 and NCAM1	0.84	83	100	0.51
EGF and NCAM1	0.91	83	100	0.59
CDH1, IGF2and EGF	1.00	100	100	0.50
CDH1, IGF2 and NCAM	0.96	91	84	0.42
CDH1, EGF and NCAM	1.00	100	100	0.50
IGF2, EGF and NCAM1	0.91	83	100	0.59
CDH1, IGF2, EGF and NCAM1	1.00	100	100	0.50

## Discussion

This study comprises in silico, systematic review of literature, and histopathology and RT-PCR sections in a quest to find potential diagnostic biomarkers and therapeutic targets for WT. Through the in silico and literature review parts of this study, a vast amount of data were analyzed using bioinformatics methods. The result was four genes and 8 genetic pathways, seemingly with utmost importance in the disease process. The 4 genes NCAM1, IGF2, EGF, and CDH1 were evaluated in terms of expression using RT-PCR on WT and control tissue samples acquired from histopathology slides of 12 WT patients. NCAM1 and IGF2 showed increased expression, while EGF and CDH1 expression was decreased. Further analysis indicated that combination of these 4 genes can discriminate WT from normal sample with 100% sensitivity and specificity.

During normal nephrogenesis, the primary NCAM1 + CD133 − multipotent stem cell differentiates into NCAM1 − CD133 + ultimate nephron cells^10^. Pode-Shakked et al. demonstrated that even though WT initiation in xenograft models was failed using less than 10,000 unsorted cells, only 500 NCAM + isolated cells resulted in tumor formation. Moreover, they eradicated WT formation by using lorvotuzumab-mertansine to inhibit NCAM1^11^. NCAM1 pro-malignant activity through FGFR receptor was shown in ovarian cancer, yet its mechanistic role in WT progression is not studied^12^. Our results were in accordance with previous reports regarding upregulated expression of NCAM1. Moreover, we did not find any significant association between different levels of NCAM1 expression and clinicopathological characteristics of patients including stage and pathologic morphology. However, this could be due to the small sample size.

The short arm of chromosome 11 plays an important role in Wilms tumorigenesis as it carries the WT1 and IGF2 genes^1^. IGF2 is a polypeptide, which belongs to the insulin family, with pivotal role in nephrogenesis as an embryonic growth factor. Physiologically, only paternal allele is expressed, and the maternal allele undergoes genomic imprinting. Any condition resulting in biallelic expression, including paternal uniparental disomy and loss of imprinting, brings about IGF2 mRNA overexpression associated with increased risk of WT, hepatoblastoma, rhabdomyosarcoma, and neuroblastoma^13–15^. IGF2 overexpression acts through PI3K and MAPK signalling pathways resulting in increased protein synthesis and cell growth and decreased apoptosis^16^. Lui et al.^17^ indicated modulation of PI3K-Akt signaling pathway via inhibition of MicroRNA (miR)-19b results in WT suppression. Also, Lou et al.^18^ suggested that miR-155-5p inhibits PI3K-Akt signaling pathway by targeting IGF2 and results in tumor suppression. Upregulated expression of IGF2 was also shown in our study. Its expression level was positively associated with male gender, while it was not associated with other clinicopathological features.

During nephrogenesis, an outpouching of the Wolffian duct named ureteric bud invades the metanephric mesenchyme and then branches. Primitive vesicles form around these branches as mesenchymal-to-epithelial transition (MET) occurs. A network of genes regulates this process including WT1^1^. WT1 operates through its various target genes, including the EGF family of growth factors, HB-EGF, AREG, and EREG. EGF family and their receptors play an important part in normal ureteric bud branching and epithelial differentiation of mesenchymal tissue^19,20^. Moreover, focal adhesion proteins such as integrins and growth factor receptors have roles in morphology, proliferation and migration of cells, and cancer cells' survival and behavior. Targeting such molecules has increased tumor sensitivities to different therapeutic modalities^21,22^.Downregulated expression seems to be associated with aberrant nephrogenesis, hence WT formation. It is reported that EGF expression has positive association with WT prognosis as its higher expression is associated with better survival^23^. However, we did not find any association between EGF expression and clinicopathological features. Small sample size could account for that.

CDH1 codes for the cell adhesion protein E-cadherin. Cell adhesion molecules (CAMs) are involved in cell–cell and cell-extracellular matrix interactions. They primarily possess tumor suppressor role. Alteration of CAMs results in tumor growth and migration due to the detachment of cells from the basement membrane. CAMs play a role in metastasis and take part in differentiation, immunological response, and signaling^24,25^. Zhang et al.^26^ indicated that inhibition of CDH1 expression through upregulated expression of miR-572 results in WT metastasis. Also, Safford et al.^27^ reported a negative association between CDH1 expression and WT metastasis. However, they did not found any association with disease recurrence. While our results demonstrated a positive significant association between CDH1 expression and male gender in WT patients, no significant association was found with other clinicopathological features. The results of ROC analysis indicate the diagnostic potential of the combined panel of the four genes: NCAM1, IGF2, EGF, and CDH1. This panel distinguished WT from normal tissue sample with 100% sensitivity and specificity. Moreover, lack of significant association between different expression levels and clinicopathological features of the disease, except for gender with IGF2 and CDH1, indicates the competency of these genes as diagnostic biomarkers. However, this study is limited by small sample size and the fact that we only had one patient with stage IV disease. Bigger sample size could reveal significant associations. Nevertheless, to define biomarkers for early disease detection, one should focus on the early stages of the disease prior to metastasis occurrence. This study is a preliminary to future research projects that struggle to validate these genes as biomarkers for WT diagnosis. The mRNAs, miRNAs, and proteins associated with these genes can also be studied to develop liquid biopsy assays to overcome invasive diagnostic methods. Several promising studies have already been done regarding this issue^28–31^.

Finally, three of eight enriched pathways were discussed along with relevant genes. Extracellular matrix (ECM) is associated with tumor cells’ shedding, progression, and invasion. ECM receptor interaction pathway is suggested as a prognostic biomarker for ductal adenocarcinoma of breast^32^. Its role in several other cancers including prostate and gastrointestinal cancers has been discussed^33–35^. Tumor cells movement throughout tissues seems to follow mechanisms analogous to leukocytes migration. Chemokines like stromal cell-derived factor-1 or CXCL12 take part in these movements^36,37^. It could be inferred that ECM receptor interaction and leukocyte transendothelial migration pathways potentially take part in WT pathogenesis. Also, Zhang et al. found several important pathways associated with WT through a semi-similar effort. Peroxisome proliferator-activated receptor signaling, and protein digestion and absorption pathways are two metabolic pathways found to be associated with WT^38^. Further evaluation and studies on these pathways could reveal novel therapeutic targets.

## Supplementary Information

Supplementary Table 1.

Supplementary Information.

Supplementary Legends.

## Data Availability

The datasets used in this paper were collected form GEO data base and literature review and are all available on www.ncbi.nlm.nih.gov. qPCR data are available per request and contact person for data request of this study is Masoumeh Majidi Zolbin. Email: mmajidizolbin@sina.tums.ac.ir , Masoumeh.majidizolbin@gmail.com.
