# Supplementary material for: The potential key genes and pathways associated with Wilms tumor in quest of proper candidates for diagnostic and therapeutic purposes
Source: Sci Rep. 2022 Oct 25;12:17906. doi: 10.1038/s41598-022-22925-3 (PMC9596724; doi:10.1038/s41598-022-22925-3)
Supplement: Supplementary file 3 — Supplementary Legends. [file 41598_2022_22925_MOESM3_ESM.docx]

**S2: List of all genes from different sources including 55 common genes**
